# Synaptic vesicle protein 2A mitigates parthanatos via apoptosis‐inducing factor in a rat model of pharmacoresistant epilepsy

**DOI:** 10.1111/cns.14778

**Published:** 2024-05-27

**Authors:** Chen Li, Ziqi Wang, Mianmian Ren, Siying Ren, Guofeng Wu, Likun Wang

**Affiliations:** ^1^ School of Clinical Medicine Guizhou Medical University Guiyang Guizhou China; ^2^ The Affiliated Hospital of Guizhou Medical University Guiyang Guizhou China

**Keywords:** apoptosis‐inducing factor, parthanatos, pharmacoresistant epilepsy, synaptic vesicle protein 2A

## Abstract

**Aims:**

Synaptic vesicle protein 2A (SV2A) is a unique therapeutic target for pharmacoresistant epilepsy (PRE). As seizure‐induced neuronal programmed death, parthanatos was rarely reported in PRE. Apoptosis‐inducing factor (AIF), which has been implicated in parthanatos, shares a common cytoprotective function with SV2A. We aimed to investigate whether parthanatos participates in PRE and is mitigated by SV2A via AIF.

**Methods:**

An intraperitoneal injection of lithium chloride‐pilocarpine was used to establish an epileptic rat model, and phenytoin and phenobarbital sodium were utilized to select PRE and pharmacosensitive rats. The expression of SV2A was manipulated via lentivirus delivery into the hippocampus. Video surveillance was used to assess epileptic ethology. Biochemical tests were employed to test hippocampal tissues following a successful SV2A infection. Molecular dynamic calculations were used to simulate the interaction between SV2A and AIF.

**Results:**

Parthanatos core index, PARP1, PAR, nuclear AIF and MIF, γ‐H2AX, and TUNEL staining were all increased in PRE. SV2A is bound to AIF to form a stable complex, successfully inhibiting AIF and MIF nuclear translocation and parthanatos and consequently mitigating spontaneous recurrent seizures in PRE. Moreover, parthanatos deteriorated after the SV2A reduction.

**Significance:**

SV2A protected hippocampal neurons and mitigated epileptic seizures by inhibiting parthanatos via binding to AIF in PRE.

## Graphical abstract

This study showed that synaptic vesicle protein 2A, manipulated by lentivirus, acts as a neuroprotective target by blocking the parthanatos pathway via binding with apoptosis‐inducing factor, ultimately improving epileptic seizures in pharmacoresistant epilepsy rats.

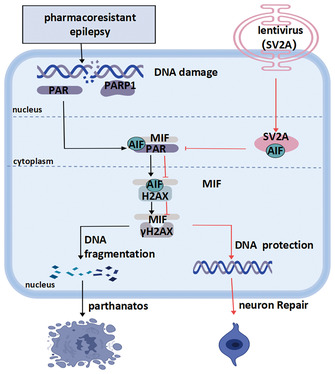

## 1 INTRODUCTION

Temporal lobe epilepsy, also known as pharmacoresistant epilepsy (PRE), is the most prevalent form of medically intractable epilepsy that responds poorly to anti‐epileptic drugs (AEDs).^1^ PRE accounts for 30%–40% of all epilepsy cases and is diagnosed clinically when two or more antiepileptic drug regimens fail to control recurring and unpredictable seizures.^2^ Institutionalized Medicare beneficiaries, the risk of injuries, and premature mortality all rose in patients with PRE due to the complicated pathogenesis of epileptogenesis.^3^ Exploring the exact mechanism behind this disorder and targeting treatment approaches remain crucial.

Synaptic vesicle protein 2A (SV2A), a transmembrane protein of synaptic vesicles implicated in the regulation of neurotransmitter release, acts as an important target of levetiracetam for the treatment of generalized epilepsy. Further, it was decreased in epileptic tissues and may even be correlated with the clinical efficacy of levetiracetam.^4^, ^5^ SV2A expression was downregulated by 30% in the hippocampus and temporal cortex of patients with pharmacoresistant temporal lobe epilepsy with hippocampal sclerosis.^6^ In some neurodegenerative and psychiatric disorders, SV2A was reduced^7^, ^8^ and SV2A loss was related to low synaptic density and brain atrophy.^9^, ^10^ Our previous study also observed a significant reduction in SV2A levels and hippocampal neuronal damage in PRE rats.^11^ Moreover, SV2A overexpression suppressed epileptic seizures and discharges in PRE.^12^

However, the relationship between SV2A loss and seizure activity is still undetermined. The expression of SV2A in neural stem cells (NSCs) was high, and SV2A knockdown could upregulate the genes involved in the p53 signaling pathway and induce NSC apoptosis.^13^ This suggests that SV2A may participate in the apoptotic signaling pathway and may play an important role in the survival regulation of neural cells. Meanwhile, levetiracetam could ameliorate mitochondrial damage in Alzheimer's disease, suggesting that SV2A may have a mitochondrial localization and protective function.^14^

As a cytoprotective protein located in the mitochondria, AIF was found to translocate to the nucleus, partially bind to DNA, and trigger large‐scale fragmentation of chromatin upon nuclear translocation.^15^ The translocation of AIF was triggered by poly ADP‐ribose (PAR) in parthanatos as well as by p53 in other apoptotic pathways.^16^, ^17^ Parthanatos, a form of programmed cell death characterized by poly (ADP‐ribose) polymerase‐1 (PARP‐1) hyperactivation and excessive synthesis of the PAR polymer, has been implicated in epileptogenesis and acute neuron injury within 24 or 48 h post‐status epilepticus in recent years^18^, ^19^; however, there have been few reports on PRE. As a key opportunity to inhibit the occurrence of parthanatos, blocking the translocation of AIF from the mitochondria to the nucleus might improve neuronal degeneration in epilepsy.^20^, ^21^ Moreover, previous research has found that AIF knockout led to aberrant NSC proliferation and defective neuronal differentiation.^22^ Given the shared protective neuronal function and mitochondrial location of SV2A and AIF, we hypothesized that SV2A can alleviate recurrent seizures of PRE by inhibiting parthanatos via interacting with AIF.

Consequently, this study aimed to determine (1) whether parthanatos is involved in PRE‐associated pathological changes and is regulated by SV2A and (2) whether and how SV2A interacts with AIF to modulate the expression of parthanatos‐related proteins and to mitigate hippocampal DNA damage.

## 2 MATERIALS AND METHODS

### 2.1 Animals

Male Sprague–Dawley rats (5 weeks old and weighing 160–180 g) were procured from Guizhou Medical University's Experimental Animal Center. The rats were housed under standard conditions with a 12‐h light–dark cycle, 20–26°C temperature, 40%–70% relatively humidity，and 15–20 lux Light intensity ≤60 dB noise. The animal experimentation protocols employed in this study were approved by the Animal Care and Use Committee of Guizhou Medical University (Guiyang, China), with approval number 2000743.

### 2.2 Epileptic models and groups

**FIGURE 1:**
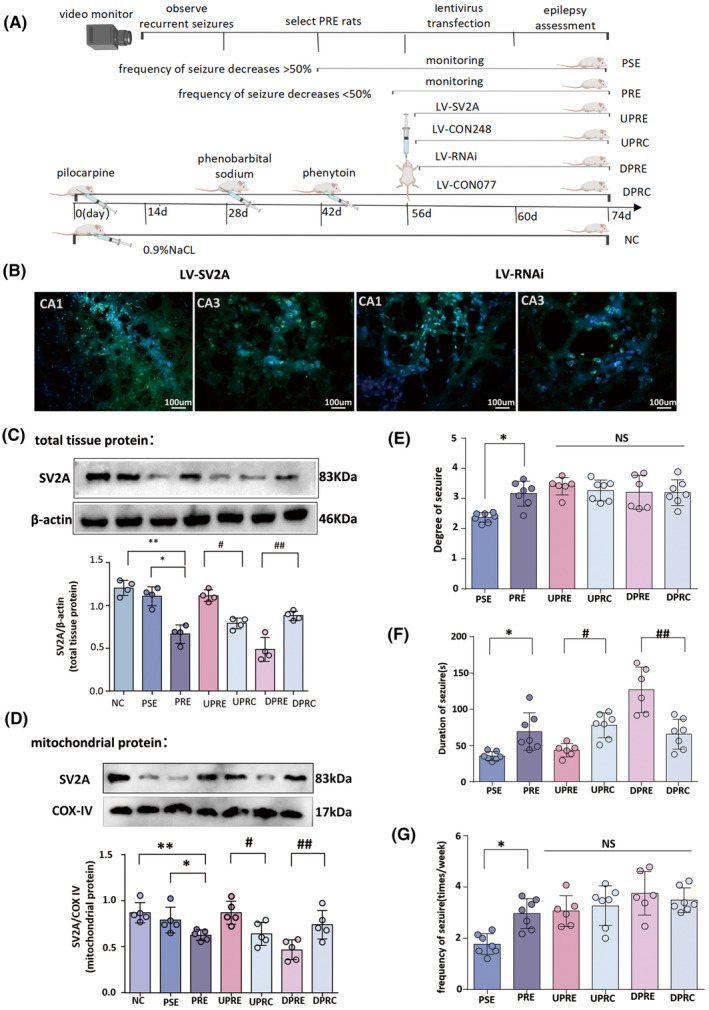
SV2A is regulated by a lentivirus and affects seizures in PRE. (A) A flowchart of epilepsy model production in this experiment and video surveillance from the 14th day of model production until the end of the 74th day; during this period, the first 28 days were used for detecting chronic epileptic rats, the next 28 days were utilized for selecting PRE rats, and the last 28 days were used for assessing the effect of SV2A on behavior. (B) Representative coronal hippocampal sections of PRE rats injected with LV‐SV2A and LV‐SV2A‐RNAi viruses. The green immunofluorescent protein (GFP, green) represents virus‐transfected cells. (C, D) SV2A expression in total protein and mitochondrial protein was detected in seven groups by western blotting. Representative blots from each group show the SV2A to β‐actin band gray value determined by a gray value analysis. SV2A expression was lower in the PRE group than in the NC and PSE groups (**p < 0.05 and *p < 0.05, respectively). SV2A expression increased in the UPRE group compared to the UPRC group and decreased in the DPRE group compared to the DPRC group. (E–G) A multivariate analysis revealing significant differences in the three seizure indices among the PSE, PRE, UPRE, UPRC, DPRE, and DPRC groups. LSD tests showed that seizure intensity, duration, and frequency were increased in the PRE group compared with the PSE group (*p < 0.001, *p < 0.001, and *p < 0.001, respectively). In the UPRE group, the seizure duration was decreased compared with the UPRC group (^#^ *p* = 0.025), while the DPRE group exhibited elevated seizure duration compared with the DPRC group (^##^ *p* = 0.026). A single asterisk indicates *p* < 0.05 compared with PSE alone. A single hashtag indicates *p* < 0.05 compared with UPRC alone, whereas two hashtags indicate *p* < 0.05 compared with DPRC alone.

In this study, 140 out of 150 Sprague–Dawley rats were used to create PRE models, whereas 10 rats were allocated to the normal control group. Two SD rats became ill and died spontaneously (NC group, *n* = 8). In 98 rats, temporal lobe status epilepticus was induced following a previously described method (lithium‐chloride pilocarpine epilepsy model).^23^, ^24^ Video surveillance was initiated 14 days after the status of epilepticus. Fifty rats, which developed seizures after 14 days of surveillance, received continuous treatment with phenobarbital sodium (PB) and phenytoin (PHT) following a previously described method for 30 days.^23^, ^24^, ^25^ Two weeks after the lentivirus was injected into the hippocampus, an assessment of the effect of SV2A on epileptic seizures started and continued for 14 days (Figure 1A). The epilepsy behavioral assessment was performed with reference to Racine scale criteria, and rats with grade III–V seizures were selected as epileptic models.^26^, ^27^ Rat brain tissues were harvested on day 74 for subsequent experiments. Among the 50 epileptic rats, five died because of frequent epilepsy, two died due to the PB treatment, and three died as a result of anesthesia.

Ultimately, 40 epilepsy model rats were categorized into six groups: a pharmacosensitive group (PSE group, *n* = 7) of epileptic rats with more than 50% decreased seizure frequency after PB and PHT treatment; a pharmacoresistant group (PRE group, *n* = 7) of epileptic rats with less than 50% decreased seizure frequency after PB and PHT treatment; an SV2A‐downregulated pharmacoresistant epileptic group (DPRE group, *n* = 6) of pharmacoresistant rats with a hippocampal injection of LV‐SV2A‐RNAi (Gene Chem); an SV2A‐upregulated pharmacoresistant epileptic group (UPRE group, *n* = 6) of pharmacoresistant rats with a hippocampal injection of LV‐SV2A (Gene Chem); an SV2A‐downregulated control epileptic group (DPRC group, *n* = 7) of pharmacoresistant rats with a hippocampal injection of LV‐CON077; and an SV2A‐upregulated control epileptic group (UPRC group, *n* = 7) of pharmacoresistant rats with a hippocampal injection of LV‐CON248 (Figure 1A).

### 2.3 Lentivirus injection and monitoring

Rats were induced into anesthesia with 5% isoflurane (RWD, Shenzhen, China) in O_2_, and their heads were securely fixed in a stereotaxic frame. In the UPRE group, a volume of 10 μL of LV‐SV2A, LV‐RNAi, LV‐CON248, or LV‐CON077 was bilaterally infused into the dorsal hippocampus (anteroposterior 3.5 mm relative to the bregma; lateral ±2.0 and 3.0 mm; dorsoventral 5.0 mm from the skull) using a microsyringe at a rate of 0.5 μL/min. The injection pipette was left in place for an additional 10–15 min to prevent backflow. Postoperatively, intraperitoneal injections of penicillin (80,000 units) were administered daily for three consecutive days. Virus transfection reached its peak after 1–2 weeks.

### 2.4 Western blot analysis

Total tissue protein, mitochondrial protein, and nuclear protein were extracted from hippocampal tissue, quantified, separated using denaturing polyacrylamide gel electrophoresis (SDS‐PAGE), and transferred onto a PVDF membrane. The membranes were incubated overnight at 4°C with the following primary antibodies: SV2A (1:1000; Abcam, USA), PAR (1:1500; Santa Cruz Biotechnology, USA), AIF (1:2000; Abcam, USA), PARP1 (1:1500; Proteintech, Wuhan, China), H2AX (1:1000; Santa Cruz Biotechnology, USA), γH2AX (1:1500; Santa Cruz Biotechnology, USA), COX‐IV (1:2500; Affinity, Jiangsu, China), Histone (1:3000; Proteintech, Wuhan, China), and β‐actin (1:7000; Proteintech, Wuhan, China). The membranes were washed with TBST and then incubated with a horseradish peroxidase‐conjugated secondary antibody for 60 min. An enhanced chemiluminescence kit was used to visualize the blots. Band density was analyzed using Image J software, and the relative band density of the target protein was normalized to β‐actin, COX IV, or histone.

### 2.5 Immunofluorescence staining

Frozen rat brain slices were blocked with a 5% BSA blocking buffer after washing with PBS at room temperature. The slices were incubated overnight with the following primary antibodies: SV2A (1:150), PAR (1:200), AIF (1:200), and γH2AX (1:150). Then, secondary antibody solutions and DAPI were diluted, pipetted onto each section, and incubated for 1 h. Images were captured using a fluorescent microscope (Olympus IX71, Japan). The total fluorescent intensities of positive cells and of DAPI staining were calculated using Image J, and a statistical analysis was performed based on the ratio of the two.

### 2.6 TUNEL staining

TDT‐mediated dUTP nick end labeling (TUNEL) was conducted according to standard protocols outlined in the manual of the one‐step TUNEL apoptosis assay kit (40306ES50, Yeasen, Shanghai, China). Apoptotic cells were double‐immunofluorescent stained for the TUNEL assay and the nuclear counterstain DAPI (blue). Subsequently, cells positively stained with green fluorescence were counted.

### 2.7 Co‐immunoprecipitation

Hippocampal tissue was collected in an immunoprecipitation buffer and analyzed by immunoblotting with an AIF mouse or rabbit anti‐Flag antibody. Proteins were separated using SDS‐PAGE and transferred to a PVDF membrane. The membrane was then blocked and incubated overnight with a 1 mg/mL rabbit anti‐Flag (anti‐SV2A, anti‐MIF, anti‐H2AX) or a mouse anti‐PAR primary antibody at 4°C, followed by a goat anti‐rabbit IgG or a donkey anti‐mouse IgG conjugated to HRP for 1 h at room temperature. After washing, immune complexes were detected using the Chemi‐luminescent system (Chemi‐scope 6200(T), CLINX, Shanghai, China).

### 2.8 Molecular docking and a dynamic simulation calculation

The crystal structures of SV2A and AIF were downloaded from the Universal Protein Data Bank (www.uniprot.org/) and modified using the HADDOCK software.^28^ The SV2A (ID:Q02563) and AIF (ID:Q9JM53) modifications included water removal, hydrogen addition, amino acid optimization, and patching.^29^ The files were saved in pdb format. AlphaFold2 was used to create 3D chemical structures and minimize energy.^30^ The SV2A and AIF structures were imported into HADDOCK, the two protein flexible keys were rotatable, and the amino acids for docking locations were finally labeled.

GROMACS 2019.6 software was used to perform molecular dynamic (MD) simulations.^31^ The system was carried out in accordance with periodic boundary conditions. First, AlphaFold2, Amber14SB, and TIP3P were used to construct an SV2A‐AIF complex model, and the parameters of the simulation system were set. Bonds were constrained by LINCS. The cut‐off for van der Waals and short‐range electrostatic interactions was set to 10 Å. Long‐range electrostatic interactions were calculated by particle‐mesh Ewald (PME). The temperature was set to 298.15 K by V‐rescale. The pressure was set to 1 Atm by isotropic Parrinello‐Rahman. Second, energy minimization was performed based on the steepest‐descent algorithm. Third, the number of particles, volume, energy (NVT), and number of particles, pressure, and temperature (NPT) phases were performed to bring the system to equilibrium. Fourth, a molecular dynamic simulation of 50 ns (a total of 25,000 steps) was performed with a time step of 2 fs, and trajectory data were saved every 10 ps. Finally, the binding free energy of the complex was calculated. Root‐mean‐square deviation of atomic positions (RMSD) analysis was performed to investigate the structural stability of the protein.^32^ Radius of gyration (*R*_g_) was performed for a protein compaction measurement.^33^ The solvent‐accessible surface area (SASA) was used to detect the molecular surface area of the SV2A‐AIF complex accessible to solvent.^34^

### 2.9 Statistical analysis

Data normality and homogeneity were assessed utilizing SPSS v26.0 (IBM, NY, USA). Data processing and graphing were performed with GraphPad Prism 8.0, and the data are presented as mean ± standard error of mean (mean ± SEM) and first tested by Shapiro–Wilk normality test. When the data follows a normal distribution，differences among the seven groups were assessed using one‐way analysis of variance (ANOVA). The least significant difference (LSD) test was employed for post hoc comparisons, and Welch's ANOVA was utilized if variances were not equal. While the differences were assessed by the Kruskal–Wallis test when the data showed a non‐normal distribution. A *p*‐value of less than 0.05 was considered statistically significant.

## 3 RESULTS

### 3.1 Regulation of SV2A expression via a lentivirus

To manipulate SV2A expression in the CA1 and CA3 regions, LV‐SV2A and LV‐SV2A‐RNAi were injected bilaterally into the polymorphic layer of the hippocampal CA2 region (located between CA1 and CA3). Fourteen days after the lentivirus injection, rats were perfused, and frozen sections were prepared for an examination. The sections revealed that green fluorescent protein was expressed in both the CA1 and CA3 regions (Figure 1B).

Western blotting was performed to assess SV2A expression in total tissue protein (SV2A‐total [*F*(6, 21) = 31.39, *p* = 0.000]) and mitochondrial protein (SV2A‐Mit [*F*(6, 28) = 5.79, *p* = 0.000]) of hippocampal tissue (Figure 1C,D). Compared to the PSE group, SV2A expression was significantly reduced in the PRE group (SV2A‐total: *p* = 0.04; SV2A‐Mit: *p* < 0.01). Following SV2A upregulation in the UPRE group, SV2A was increased compared to the UPRC group (SV2A‐total: *p* = 0.001; SV2A‐Mit: *p* = 0.04). Conversely, following SV2A downregulation in the DPRE group, SV2A expression was decreased significantly compared with the DPRC group (SV2A‐total: *p* < 0.001; SV2A‐Mit: *p* < 0.001).

### 3.2 SV2A upregulation reduced seizures

Significant differences in the spontaneous seizure degree ([*F*(5, 34) = 6.28, *p* = 0.00]; Figure 1E), duration (seconds; [*F*(5, 34) = 15.90, *p* = 0.00]; Figure 1F), and frequency (times per week; [*F*(5, 34) = 8.27, *p* = 0.00]; Figure 1G) were observed among the groups. Compared to the PSE group, the seizure degree (*p* < 0.001), frequency (*p* < 0.001), and duration (*p* < 0.001) were significantly increased in the PRE group. Following SV2A upregulation, the seizure duration was significantly reduced in the UPRE group compared with the UPRC group (46.98 ± 12.42 vs. 77.88 ± 17.18, *p* = 0.025). However, after SV2A was downregulated in the DPRE group, the seizure duration was significantly elevated compared to the DPRC group (126.79 ± 31.31 vs. 65.73 ± 20.71, *p* = 0.026). These results suggest that SV2A plays an important role in controlling epileptic seizures. The degree and frequency of epilepsy, however, showed no significant differences among the five pharmacoresistant groups (Figure 1E and 1G).

### 3.3 SV2A upregulation inhibited the hyperactivation of PARP1, PAR, and MIF

**FIGURE 2:**
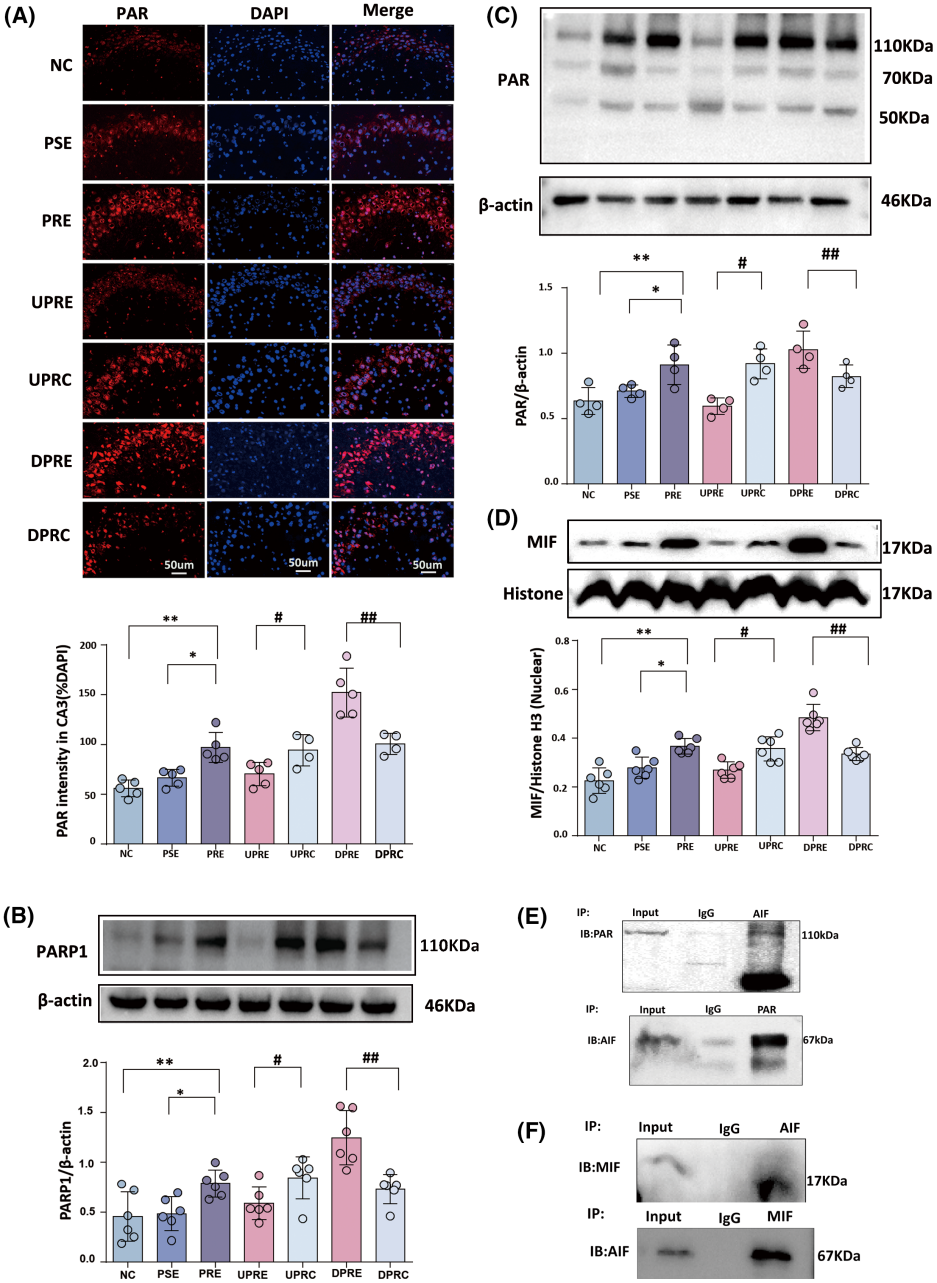
SV2A regulation alters the expression of PARP1, PAR, and MIF. (A) Representative micrographs demonstrate PAR expression in the CA3 regions of the seven groups. Total fluorescence intensity ratio of PAR. (B, C) Immunoblotting analysis of PARP‐1 and PAR polymers after SV2A regulation. Band grayscale value ratios of PARP‐1 or PAR to β‐actin. (D) Immunoblotting analysis of MIF. Band grayscale value ratios of MIF to Histone H3. (E, F) Immunoblotting shows co‐expression of AIF and PAR or AIF and MIF when the two proteins are used as flag proteins for immunoprecipitation. The results are presented as mean ± SEM; a single asterisk indicates *p* < 0.05 compared with PSE alone, whereas two asterisks indicate *p* < 0.05 compared with NC alone. A single hashtag indicates *p* < 0.05 compared with UPRC alone, whereas two hashtags indicate *p* < 0.05 compared with DPRC alone. MIF, migration inhibitory factor; PARP‐1, poly (ADP‐ribose) polymerase‐1.

PAR was expressed as red fluorescence in both the nucleus and cytoplasm of CA3. Weak fluorescence was detected in the NC group (Figure 2A). There were significant differences in the immunofluence intensity of PAR among seven groups [*F*(6, 26) = 15.750, *p* = 0.000]. Compared with the PSE group, the fluorescence intensity ratio of PAR was increased in the CA3 area (*p* = 0.01). Following SV2A upregulation in the UPRE group, the fluorescence intensity ratio of PAR was reduced compared with the UPRC group (*p* = 0.01). Following SV2A downregulation in the DPRE group, the fluorescence intensity ratio of PAR was increased compared with the DPRC group (*p* = 0.004).

There were significant differences in PARP1 ([*F*(6, 35) = 11.12, *p* = 0.000], Figure 2B), PAR ([*F*(6, 21) = 8.99, *p* = 0.000], Figure 2C), and MIF ([*F*(6, 35) = 23.66, *p* = 0.000], Figure 2D) among the seven groups. Compared with the PSE group, there were increased PARP1 (*p* = 0.03), PAR (*p* = 0.02), and MIF (*p* = 0.001) in the PRE group. Decreased PARP1 (*p* = 0.02), PAR (*p* < 0.001), and MIF (*p* = 0.001) were detected after SV2A upregulation (in the UPRE group) compared to the UPRC group. Elevated PARP1 (*p* = 0.04), PAR (*p* = 0.01), and MIF (*p* = 0.001) were detected after SV2A downregulation (in the DPRE group) compared to the DPRC group.

High affinity was detected between AIF and PAR (Figure 2E) and AIF and MIF (Figure 2F) using co‐immunoprecipitation of total tissue protein in the PRE group.

### 3.4 SV2A upregulation inhibited the nuclear translocation of AIF

**FIGURE 3:**
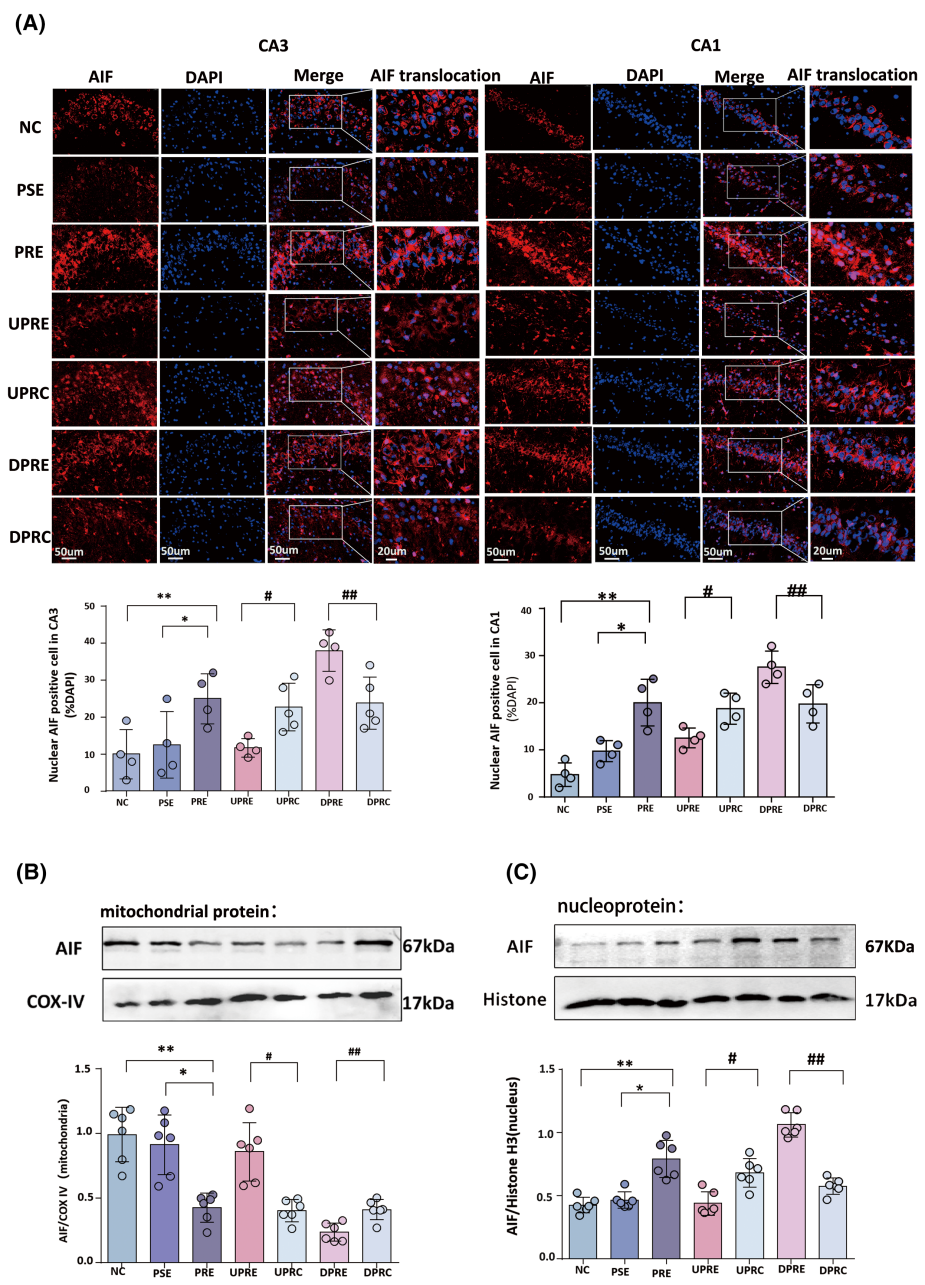
SV2A regulation affects the nuclear translocation of AIF. (A) Representative micrographs demonstrate AIF expression in the CA1 and CA3 regions of the seven groups. Scale bar: 50 μm, and high magnification with 20 μm for AIF translocation. The ratio of nuclear AIF‐positive cells determined by a fluorescence intensity analysis is shown in the histogram. (B, C) Mitochondrial and nuclear AIF levels after SV2A regulation by western blotting. AIF to COX IV and AIF to histone were determined by a grayscale value analysis. The results are presented as mean ± SEM; a single asterisk indicates *p* < 0.05 compared with PSE alone, whereas two asterisks indicate *p* < 0.05 compared with NC alone. A single hashtag indicates *p* < 0.05 compared with UPRC alone, whereas two hashtags indicate *p* < 0.05 compared with DPRC alone. AIF, apoptosis inducing factor.

AIF was expressed as red fluorescence in both the nucleus and cytoplasm of CA1 and CA3 in the PRE‐related groups (Figure 3A). Cells were defined as AIF‐positive if the area of AIF nuclear expression encompassed more than half of the DAPI area. There were significant differences in nuclear AIF‐positive in both CA1 [*F*(6, 21) = 11.45, *p* = 0.000] and CA3 [*F*(6, 23) = 9.22, *p* = 0.000] areas. Compared with the PSE group, the AIF‐positive cell ratio was increased in the PRE group (CA3: 25 ± 6.78% vs. 12.5 ± 9%, *p* = 0.01; CA1: 22.5 ± 9.11% vs. 9.75 ± 2.22%, *p* = 0.002). Following SV2A upregulation in the UPRE group, the AIF‐positive cell ratio (11.75 ± 2.5% vs. 22.8 ± 6.46% in CA3, *p* = 0.02; 12.75 ± 1.71% vs. 20.5 ± 5.80% in CA1, *p* = 0.04) was reduced compared with the UPRC group. Following SV2A downregulation in the DPRE group, the AIF‐positive cell ratio was increased compared with the DPRC group (38.00 ± 5.6% vs. 23.8 ± 7.05% in CA3, *p* = 0.004; 30 ± 5.16% vs. 20.25 ± 4.79% in CA1, *p* = 0.01).

Western blotting was performed to detect the expression of AIF in mitochondrial protein ([*F*(6, 35) = 21.65, *p* = 0.000], Figure 3B) and nuclear protein ([*F*(6, 35) = 35.26, *p* = 0.000], Figure 3C). There were significant differences in AIF expression among the seven groups (*p* < 0.001). Compared with the PSE group, there was increased AIF expression in the nucleus (*p* = 0.025) and decreased AIF expression in the mitochondria (*p* = 0.006) in the PRE group. SV2A upregulation was associated with elevated AIF levels in the mitochondria (*p* = 0.025) and significantly reduced AIF levels in the nucleus (*p* = 0.03) compared to the UPRC group. However, SV2A downregulation was characterized by reduced AIF in the mitochondria (*p* = 0.022) and increased AIF in the nucleus (*p* < 0.001).

### 3.5 SV2A bound to AIF to form a stable complex

**FIGURE 4:**
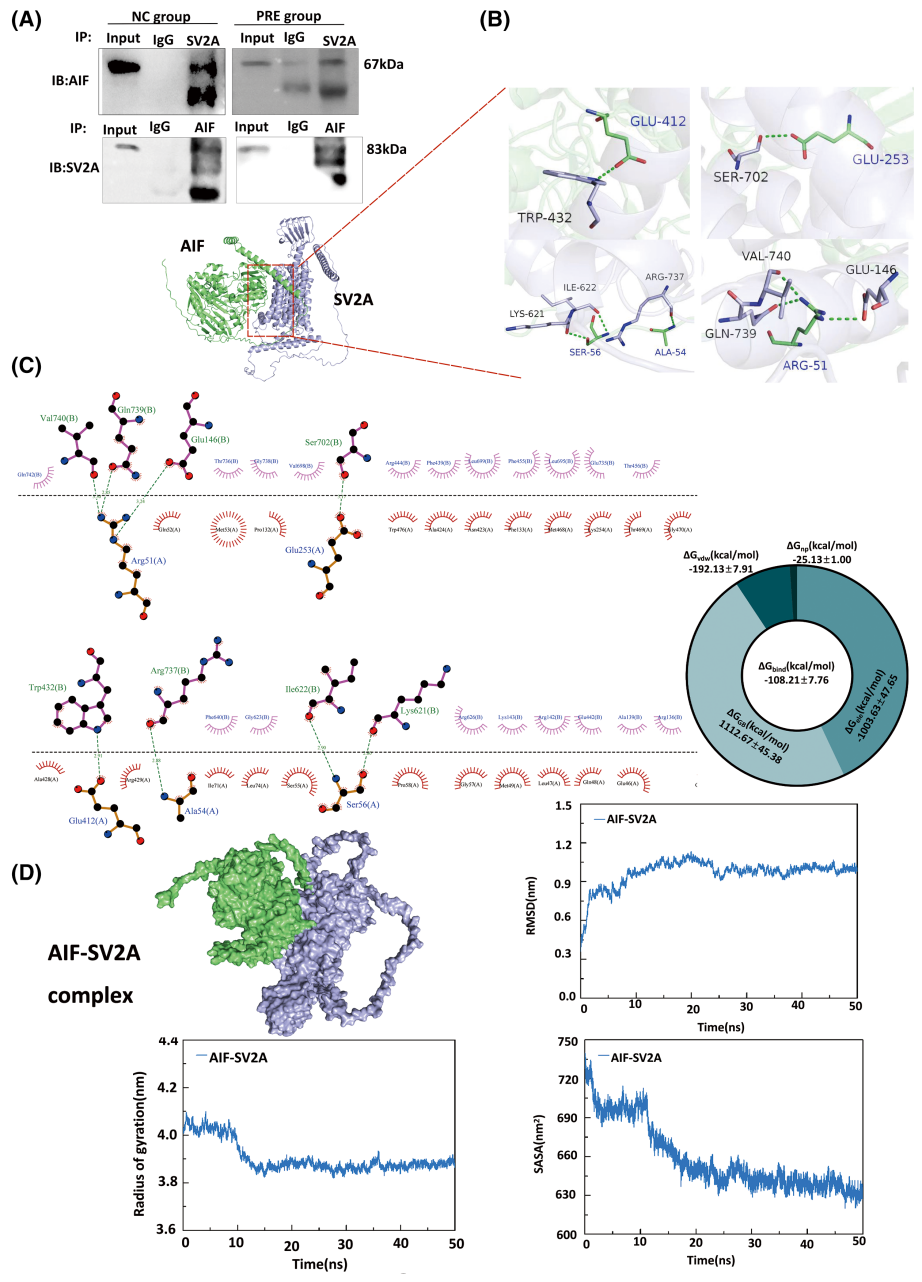
SV2A binds to AIF to form structurally stable complexes. (A) Immunoblotting shows co‐expression of AIF and SV2A when the two proteins are used as flag proteins for immunoprecipitation. (B) The docking sites of amino acids for AIF and SV2A binding and complex formation. (C) The interaction between major linking amino acids and amino acid residues in the SV2A‐AIF complex, with green dashed lines representing hydrogen bonds, and eyelash shapes representing hydrophobic bonds. The circular diagram shows the various binding energies of the AIF‐SV2A complex, including the van der Waals energy term (ΔGvdw), electrostatic energy term (ΔGele), polar solvation energy (ΔGGB), and nonpolar solvation energy (ΔGnp), with the total binding energy (ΔGbind) at the center of the ring. (D) The stability of the SV2A‐AIF complex, change of the root mean square deviation (RMSD), and gyration solvent accessible surface area; all these three indexes stabilized at 20 ns.

In the NC and PRE groups, mitochondrial proteins were used to examine the interaction between AIF and SV2A via co‐immunoprecipitation. High affinity between SV2A and AIF was observed in both groups (Figure 4A).

We identified three main structural domains of AIF (from the N‐ to C‐terminus): mitochondrial localization signal (MLS) domain (amino acids 1–62), pyridine nucleotide disulphide oxidoreductase (PNDO) domain (amino acids 135–460), and mitochondrial‐associated C‐term domain (amino acids 464–594). We identified the SV2A binding site in AIF, where ARG^51^, ALA^54^, and SER^56^ reside within the MLS domain, and GLU^412^ and GLU^253^ reside within the PNDO domain. Based on the simulated reconstruction structure, the model of SV2A included 12 helical transmembrane domains, seven cytoplasmic domains, and six extracellular domains. Among these SV2A domains, we identified five AIF binding sites, with SER^702^ residing within one out of 12 helical domains and GLU^146^, TRP^432^, LYS^621^‐ILE^622^, and ARG^737^‐GLN^739^‐VAL^740^ residing within four different cytoplasmic domains (Figure 4B).

The RMSD curve represents fluctuations in protein conformation. The RMSD of the SV2A‐AIF complex has certain fluctuations in the early stage but tends to stabilize after 20 ns, indicating that after SV2A binds to AIF, the conformation of AIF does not significantly change, and the combination of both is relatively stable (Figure 4D). The gyration radius curve represents the compactness of the overall structure of the protein. The gyration radius of the SV2A‐AIF complex is stable. SASA decreased continuously with time and finally stabilized at about 630 nm. These results were consistent with the RMSD findings, indicating that the protein conformation was stable and compactly folded (Figure 4D). In addition, we calculated the binding free energy of the RMSD stable phase (30–50 ns) of the SV2A‐AIF complex, and the result was −108.21 kcal/mol. The van der Waals potential (−192.13 kcal/mol), electrostatic interaction (−1003.63 kcal/mol), and nonpolar solvation free energy (−25.13 kcal/mol) were conducive to the combination of the two, while the polar solvation energy (1112.67 kcal/mol) was not conducive to their interaction (Figure 4C).

### 3.6 SV2A upregulation inhibited AIF‐mediated H2AX phosphorylation and DNA fragmentation

**FIGURE 5:**
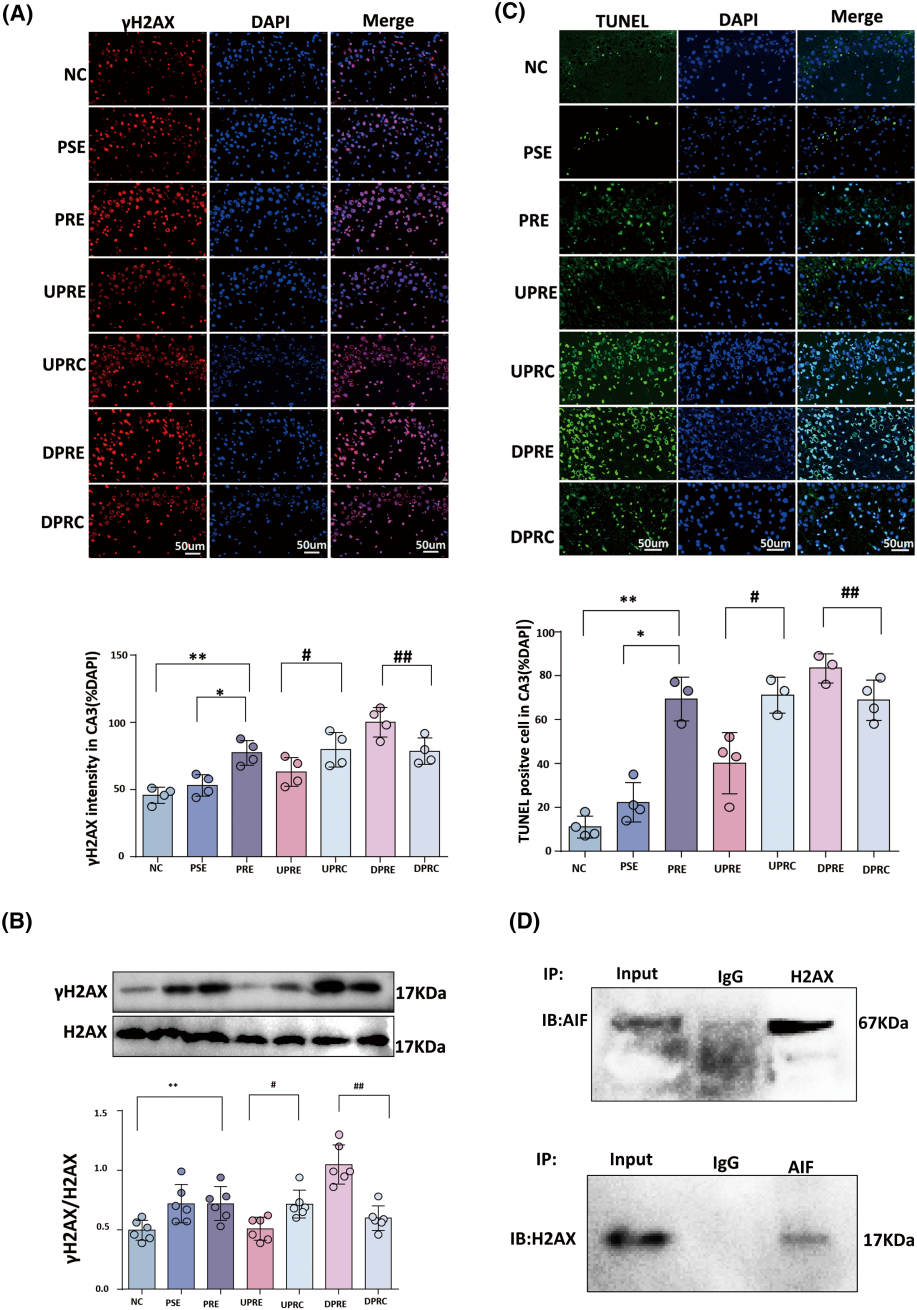
SV2A upregulation reduces γH2AX expression and DNA fragmentation. (A) Representative micrographs show γH2AX staining in the CA3 regions of the seven groups. Scale bar: 50 μm. The total fluorescence intensity ratio of γH2AX to DAPI was determined by a fluorescence intensity analysis. (B) Representative immunoblots of γH2AX and H2AX expression in each group. The band grayscale value ratios of γH2AX to H2AX determined by a grayscale value analysis are presented in the histogram. (C) Representative micrographs of TUNEL staining in the CA3 regions and the ratio of TUNEL‐positive cells in the seven groups. The rate of TUNEL‐positive cells was increased in the PRE group compared with the PSE group, decreased in the UPRE group, and increased in the DPRE group. The results are presented as mean ± SEM; a single asterisk indicates *p* < 0.05 compared with PSE alone, whereas two asterisks indicate *p* < 0.05 compared with NC alone. A single hashtag indicates *p* < 0.05 compared with UPRC alone, whereas two hashtags indicate *p* < 0.05 compared with DPRC alone. (D) Co‐expression of AIF and H2AX when used as a bait protein and a flag protein for immunoprecipitation and immunoblotting.

γH2AX was expressed as red fluorescence in the nucleus, and the different significances existed among seven groups ([*F*(6, 21) = 13.99, *p* = 0.000], Figure 5A). Compared with the PSE group, the fluorescence intensity ratio of γH2AX was increased in the CA3 area of the PRE group (*p* = 0.002). The fluorescence intensity ratio of γH2AX was decreased in the UPRE group compared with the UPRC group (*p* = 0.03). The fluorescence intensity ratio of γH2AX was increased in the DPRE group compared with the DPRC group (*p* = 0.005). Western blotting showed different significances existed among seven groups [*F*(6, 35) = 12.84, *p* = 0.000]. γH2AX expression increased in the PRE group compared to the NC group (*p* = 0.005; Figure 5B). SV2A upregulation in the UPRE group was associated with a decreased γH2AX level compared to the UPRC group (*p* = 0.008). Conversely, the DPRE group exhibited an increased γH2AX level compared with the DPRC group (*p* < 0.001; Figure 5B).

A TUNEL assay was employed to detect DNA strand break fragments. There were statistically significant differences among the seven groups in the CA3 area ([*F*(6, 18) = 37.34, *p* = 0.000], Figure 5C). In the NC group, the TUNEL‐positive cell ratio was 19 ± 10.23%, and it was increased in the PRE group compared with the PSE group (69.33 ± 10.02% vs. 19.75 ± 4.57%, *p* < 0.001) and in the DPRE group compared with the DPRC group (83.33 ± 6.66% vs. 68.75 ± 9.18%, *p* = 0.04). Compared with the UPRC group, the TUNEL‐positive cell ratio was reduced in the UPRE group (40 ± 13.88% vs. 71 ± 8.19%, *p* < 0.001).

High affinity was detected between AIF and H2AX in total tissue protein from the PRE group via co‐immunoprecipitation (Figure 5D).

## 4 DISCUSSION

This study demonstrated the effect of SV2A regulation on parthanatos‐related indices in PRE models and resulted in two key discoveries. First, parthanatos participated in hippocampal neuronal injury and epileptic progression in PRE. Second, SV2A acted as a neuroprotective factor in parthanatos via binding with AIF.

Parthanatos was first demonstrated to occur in PRE rats and involved PARP‐1 hyperactivation, accumulation of PAR, binding of PAR and AIF, release of AIF from the mitochondria, translocation of the AIF/MIF complex, and MIF and γ‐H2AX‐mediated DNA fragmentation. Previous research has indicated that parthanatos occurs within 48 h after epilepsy and glutamine‐induced cell injury.^19^ Our study showed that, if parthanatos was mitigated, epileptic seizures were also prevented, suggesting that parthanatos is not merely a consequence of epilepsy but a key contributor to epileptogenesis in PRE. A better understanding of the molecular mechanism of parthanatos may provide a new approach to the treatment of PRE, and core proteins in parthanatos are also expected to become a therapeutic target for PRE.^35^

As the initiating factor of parthanatos, PARP1 is of considerable interest for targeting its therapeutic activity and is inhibited by upregulating SV2A in our study. As a matter of fact, inhibition of PARP‐1 has been recognized as a beneficial strategy for epilepsy.^20^ Small interfering RNA‐mediated PARP‐1 knockdown could effectively protect HT22 cells against glutamate‐induced toxic effects.^19^ The neuroprotective effect of TRPM2 knockout could decrease neuronal apoptosis via PARP‐1/BNIP3/AIF in epilepsy.^36^ KIF4 mutations increased the affinity of binding with PARP1 and contributed to epilepsy susceptibility.^37^

In this study, SV2A protected mitochondria, inhibiting the translocation of AIF to the nucleus, subsequently inhibiting parthanatos, and ultimately protecting DNA from degradation. As a unique target for treating epilepsy, molecular‐level alterations of SV2A have been predicted to affect susceptibility to pharmacoresistance.^38^ Previous research demonstrated that a SV2A mutant may be a key epileptogenic step in the mammalian system.^39^ However, the mechanism via which SV2A loss translates into epilepsy and pharmacoresistance remains unclear. Our results suggested that SV2A may act as an anti‐apoptotic factor via its interactions with AIF. In fact, evidence from previous studies also supported the protective effect of SV2A on mitochondria. Based on the similarity between mitochondrial and vesicular membranes, SV2A has been proposed to act as a fusion or fission factor for mitochondria in SH‐SY5Y cells.^14^ Levetiracetam, which is an SV2A ligand, has demonstrated neuroprotective effects by affecting mitochondrial function and permeability.^14^ Administration of levetiracetam after continuing seizures could significantly reduce glutathione and protect against mitochondrial dysfunction.^40^ Another study demonstrated an inhibitory effect of levetiracetam on the opening of the mitochondrial permeability transition pore.^41^

More specifically, AIF has been demonstrated as the crux for SV2A's effect on parthanatos, and SV2A may act as a structurally stable protective factor for AIF. The amino acids responsible for the interaction between SV2A and AIF were detected through molecular docking; among them, Met^54^ and Ala^55^ were the key amino acids that promoted the structural change of AIF.^42^ Molecular dynamic simulation showed that protein conformation became more compact and stable after docking of SV2A and AIF. AIF has been demonstrated to play a centric role in apoptosis pathways, including parthanatos. We speculated that AIF might undergo an allosteric change due to SV2A binding and might therefore reduce migration to the cytoplasm as well as to the nucleus. This may be the key point by which SV2A regulates the parthanatos.

**FIGURE 6:**
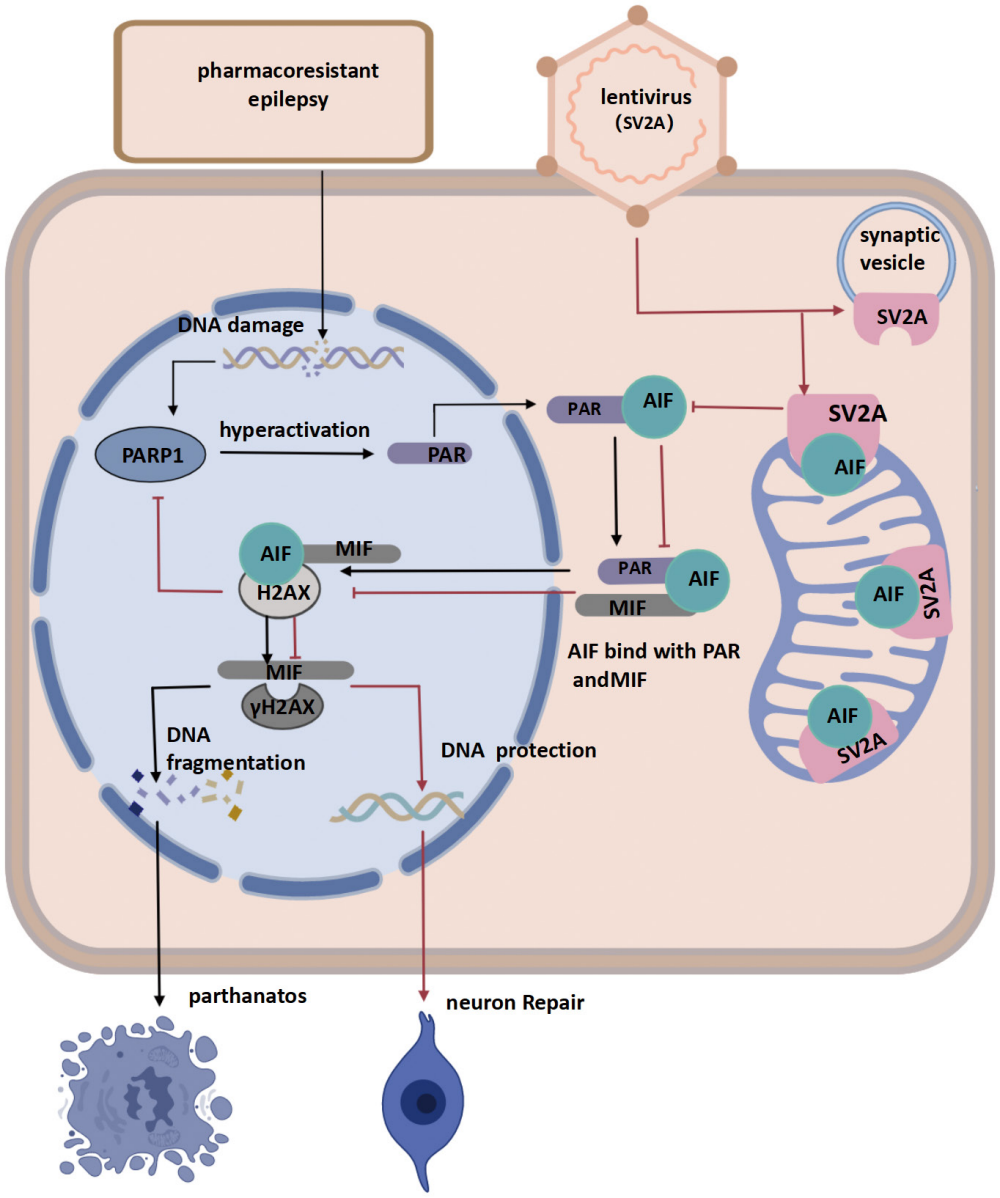
The binding of SV2A‐AIF mitigates the parthanatos pathway. A schematic model proposing a mechanism via which SV2A mitigates parthanatos. The black arrows represent the classic parthanatos process: DNA damage leads to PARP1 activation, nuclear protein PARylation and cytoplasmic translocation, binding to fragmented AIF in the mitochondria, and nuclear translocation, leading to DNA fragmentation upon H2AX binding. The red arrows represent the process of SV2A inhibition of parthanatos: SV2A binds to AIF to inhibit its translocation from the mitochondria, subsequently inhibiting AIF nuclear translocation, PAR activation, and DNA fragmentation, and thereby protecting DNA and reducing recurrent epileptic seizures.

Upon combination of AIF and SV2A, there was a reduction of AIF truncation and DNA degradation in PRE. AIF induces DNA fragmentation via two factors (Figure 6). One factor was parthanatos‐associated AIF nuclease (PAAN), also known as MIF. MIF was recruited by AIF and transferred into the nucleus, where it cleaved genomic DNA into large‐scale fragments.^43^ The function of MIF in Parkinson's disease has been elucidated, but only a few studies have been conducted in PRE.^44^ The other factor was H2AX, which was recruited by AIF in the nucleus and transformed into its phosphorylated form (γH2AX) when DNA was severely damaged, particularly in cases of DNA double‐strand breaks.^45^ Considering the interaction between AIF and H2AX, γH2AX might serve as a signal indicator for the influence of AIF on DNA injury. The inhibitory effect of SV2A on MIF and γH2AX was also demonstrated, suggesting that SV2A might inhibit DNA injury and cell apoptosis through AIF, thus affecting the survival of neurons and reversing epileptogenesis. Consequently, our results helped elucidate the mechanism via which SV2A improves neuronal function in PRE.

In this study, we demonstrated for the first time exacerbated parthanatos in PRE rats, shedding new light on the mechanisms involved in PRE. Epileptic seizures in rats were observed via video recordings, and the subjective judgment of observers regarding the seizure grade and duration may have introduced variability. Additionally, due to the relatively high mortality rate associated with the employed modeling method, issues such as drug resistance in drug screening led to a small sample size of pharmacoresistant rats, potentially introducing a bias into the experimental results.

## 5 CONCLUSION

The present study demonstrated that SV2A acts as a neuroprotective factor by blocking the parthanatos pathway via binding with AIF, ultimately improving epileptic seizures in PRE.

## AUTHOR CONTRIBUTIONS

In this article, Chen li and Ziqi Wang are the first authors and contributed equally to this work Chen Li was responsible for the overall arrangement and quality control of the experiments; Mianmian Ren was responsible for the production of the pharmacoresistant epilepsy model; Ziqi Wang was responsible for performing the western blot and immunofluorescence experiments; Siying Ren and Guofeng Wu provided suggestions for the statistical analysis of the experiments and writing of the article; Likun Wang conceptualized the experiments and provided funding for the entire study.

## FUNDING INFORMATION

This research was supported by the Natural Science Foundation of China (82001380), Guizhou Provincial Science and Technology Projects Qiankehe platform talent [2021 general (5612)], the Cultivation Program of the National Natural Science Foundation and Leading Talents in the Department, and the Affiliated Hospital of Guizhou Medical University (gyfynsfc‐2021‐8, gyfynsfc‐2021‐18, and gyfyxkyc‐2023‐05).

## CONFLICT OF INTEREST STATEMENT

None of the authors has any conflict of interest to disclose.

## Data Availability

The data that support the findings of this study are available from the corresponding author upon reasonable request.
